# Investigation of a real-time EPID-based patient dose monitoring safety system using site-specific control limits

**DOI:** 10.1186/s13014-016-0682-y

**Published:** 2016-08-12

**Authors:** Todsaporn Fuangrod, Peter B. Greer, Henry C. Woodruff, John Simpson, Shashank Bhatia, Benjamin Zwan, Timothy A. vanBeek, Boyd M.C. McCurdy, Richard H. Middleton

**Affiliations:** 1 Faculty of Engineering and Built Environment, School of Electrical Engineering and Computer Science, University of Newcastle, Newcastle, NSW Australia; 2 Faculty of Science and IT, School of Mathematical and Physical Sciences, The University of Newcastle, Newcastle, NSW Australia; 3 Radiation Physics Laboratory Sydney Medical School, The University of Sydney, Sydney, NSW Australia; 4 Calvary Mater Newcastle Hospital, Newcastle, NSW Australia; 5 Central Coast Cancer Centre, Gosford, NSW Australia; 6 Division of Medical Physics, CancerCare Manitoba, Winnipeg, MB Canada; 7 Department of Physics and Astronomy, University of Manitoba, Winnipeg, MB Canada; 8 Department of Radiology, University of Manitoba, Winnipeg, MB Canada

**Keywords:** Statistical process control, Real-time patient dose monitoring safety system, EPID

## Abstract

**Purpose:**

The aim of this study is to investigate the performance and limitations of a real-time transit electronic portal imaging device (EPID) dosimetry system for error detection during dynamic intensity modulated radiation therapy (IMRT) treatment delivery. Sites studied are prostate, head and neck (HN), and rectal cancer treatments.

**Methods:**

The system compares measured cumulative transit EPID image frames with predicted cumulative image frames in real-time during treatment using a χ comparison with 4 %, 4 mm criteria. The treatment site-specific thresholds (prostate, HN and rectum IMRT) were determined using initial data collected from 137 patients (274 measured treatment fractions) and a statistical process control methodology. These thresholds were then applied to data from 15 selected patients including 5 prostate, 5 HN, and 5 rectum IMRT treatments for system evaluation and classification of error sources.

**Results:**

Clinical demonstration of real-time transit EPID dosimetry in IMRT was presented. For error simulation, the system could detect gross errors (i.e. wrong patient, wrong plan, wrong gantry angle) immediately after EPID stabilisation; 2 seconds after the start of treatment. The average rate of error detection was 7.0 % (prostate = 5.6 %, HN= 8.7 % and rectum = 6.7 %). The detected errors were classified as either clinical in origin (e.g. patient anatomical changes), or non-clinical in origin (e.g. detection system errors). Classified errors were 3.2 % clinical and 3.9 % non-clinical.

**Conclusion:**

An EPID-based real-time error detection method for treatment verification during dynamic IMRT has been developed and tested for its performance and limitations. The system is able to detect gross errors in real-time, however improvement in system robustness is required to reduce the non-clinical sources of error detection.

## Background

In vivo dosimetry using an electronic portal imaging device (EPID) has been investigated and implemented clinically to improve the quality of treatment and safety during external beam radiotherapy (EBRT) [1–3]. This has been shown to be of particular benefit to modern dynamic delivery techniques such as intensity modulated radiation therapy (IMRT) and volumetric modulated arc therapy (VMAT) as well as for hypo-fractionated deliveries, for example stereotactic body radiation therapy (SBRT). These dynamic delivery techniques involve the delivery of non-intuitive fluences and multi-leaf collimator (MLC) trajectories as well as dose rate and, in the case of VMAT, gantry rotation modulations in order to achieve a highly conformal three-dimensional dose distribution within the patient. Due to the added complexity, pre-treatment quality assurance (QA) for EBRT has been the focus of many recent studies [4–8]. However, there is still the potential for random and systematic delivery uncertainties, at each fraction of the treatment course. Traditional pre-treatment QA is unable to detect these types of errors which are unique to each fraction, for example, patient anatomy changes, undetected machine errors, inadequate immobilisation and human error (e.g. accidental plan modification, incorrect treatment site or plan/patient, etc.) [2, 9, 10].

The Netherlands Cancer Institute have fully replaced pre-treatment verification with 3D EPID-based in vivo dosimetry. In their system, the delivered dose is verified offline by comparing the planned and reconstructed 3D dose distributions using a gamma evaluation with a 3 %/3 mm acceptance criteria [11]. Three key parameters, mean gamma (γ_mean_), 99^th^ percentile gamma (γ_1%_), and dose different at isocenter (ΔD_iso_), were employed to detect dose delivery errors. They defined the tolerance levels as γ_mean_ > 0.5, γ_1%_ > 2, and ΔD_iso_ > 3 %, and the action levels were γ_mean_ > 1, γ_1%_ > 4, and ΔD_iso_ > 5 % [5]. This work has demonstrated that offline in vivo EPID-based dosimetry has the ability to provide clinically useful information regarding the accuracy of the dose delivered to each patient at each individual treatment fraction. It has also been shown that, using automation, this can potentially be less time consuming than traditional phantom-based pre-treatment QA.

Whilst offline in vivo dosimetry does improve the ability to detect delivery errors, it is still limited in its capacity to prevent errors before clinically significant errors occur [12]. This is of particular significance for hypo-fractionated deliveries, where detected errors cannot be compensated for in subsequent fractions. Real-time in vivo EPID dosimetry has the potential to not only detect, but also to prevent clinically significant dose delivery errors. We have developed the first system where transit EPID images frames are acquired and compared to predicted image frames in real-time during the delivery of dynamic EBRT [13, 14]. In this system a 4 %/4 mm Chi (χ) comparison (a computationally efficient implementation of the γ comparison [11]) was used to compare (a) each individual frame of the measured and predicted images (b) the cumulative measured and predicted image frames. Previously, an arbitrary low threshold level was used with the aim of detecting gross delivery errors (50 % pass rate for the frame-by-frame comparison and 60 % for the cumulative comparison). An appropriate threshold level for optimal error detection using real-time transit EPID dosimetry has not yet been determined.

Statistical process control (SPC) techniques have been introduced for QA in radiation therapy, allowing for a more rigorous approach to monitoring variation within a system [15, 16]. SPC has the ability to separate systematic and random errors within a system of measurements, while the traditional methods, using standard deviation cannot. For example, Gerard et al. [17] applied SPC to detect significant random variations and used performance capability indices to evaluate pre-treatment IMRT QA for head-and-neck and prostate plans. This methodology can be applied to the analysis of real-time transit EPID dosimetry where historical treatment data can be used to detect errors (i.e. significant changes in the system) during the treatment. The term “error” is used loosely here to represent any treatment deviation that is detected by the system including those due to random and systematic anatomical changes in the patient, machine malfunctions and real-time verification system failures.

In this paper treatment site-specific control limits for error detection have been developed based on measured transit EPID image frames and predictions for a set of 137 patients using 274 measured treatment fractions. These control limits were developed to evaluate the delivery and can be applied either in real-time or off-line (i.e. after the fraction has been delivered). The aim is to enable a systematic approach to error detection and error classification and to assist with development of the real-time transit EPID dosimetry system by quantifying its performance and its limitations.

## Materials and methods

### Data collection and selection method

The real-time patient treatment verification system, known as “Watchdog”, was initially operated passively during patient treatments to acquire transit EPID images. No interventional action, or otherwise, was performed during the course of the patients treatment. This study was approved by the local human research ethics committee. Collection of data was divided into two phases: 1) training data collection for determining lower control limits (i.e. thresholds or action levels), and 2) data collection for evaluating the system performance.

To determine lower control limits (LCL) (refer to section 2.3), the first two fractions were used for all patients as these were closest in time to the reference condition (simulation) and therefore less likely to experience anatomical change such as weight loss and tumour shrinkage. A similar assumption has been made in other studies where the measured EPID images acquired from the first fraction were used as a reference data set for comparison throughout the following fractions [12]. The selected data was used as “training data” and thus it was verified that there were no substantial errors in either the delivery, acquisition or the plan generation. Three key constraints were used to select the training data to ensure this. Initially, the first two fractions were selected to determine the LCL. Secondly, any fields with final cumulative χ pass rate (i.e. the pass-rate for the integrated field) less than 97 % were also excluded. Thirdly, any data containing known Watchdog related system errors and/or human errors (e.g. incorrect image acquisition process) were also excluded. These constraints resulted in the training data consisting of 137 patient treatment courses (18 rectum patients, 82 prostate patients, and 37 HN patients). All treatments were planned using Eclipse (Varian Medical Systems, Palo Alto, CA) version 11.

### Watchdog: real-time EPID-based patient dose monitoring safety system

#### Delivery system and image acquisition

All treatments were performed using one of four Clinac Trilogy Linear Accelerators (linacs) (Varian Medical Systems, Palo Alto, CA) equipped with Millenium 120-leaf multi-leaf collimators (MLCs). Delivery was dynamic sliding-window IMRT using 6 MV photons at nominal dose rates of 400 MU/min. Megavoltage (MV) images were acquired using an aS1000 EPID operating in integrated acquisition mode controlled by the clinical treatment software module within the 4D Integrated Treatment Console (4DITC) PC. All images were automatically dark field and flood field corrected and were acquired with a source-to-detector distance (SDD) of 150 cm. Access to both MV and kV individual image frames was via camera-link cables to ports on the 4DITC and Varian On-Board Imager (OBI) computers. These were connected to an ancillary PC equipped with a dual-base frame-grabber card (Matrox Solios SOL 2 M EV CLB). Gantry rotation angles were derived from kilovoltage (kV) source rotation information encoded in the header of kV “dark” image frames by the Varian OBI. Note that the kV source was *not* on during acquisition of the kV frames. Patients received no additional dose from this study. The raw frame grabber data was reconstructed into matrix image format using an in-house custom Matlab/C# (MathWorks, Natick, MA, USA) code. This system acquired both MV and kV image frames at frame rates of 7.455 fps and 10.92 fps, respectively.

#### Overview of watchdog system

**Fig. 1:**
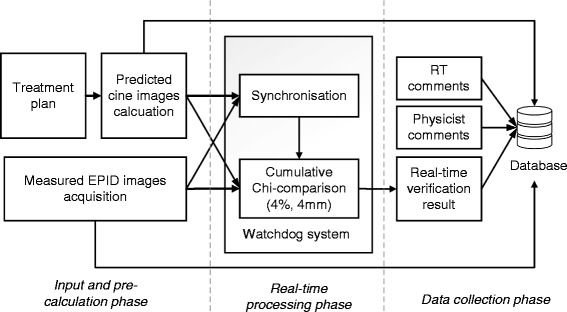
Watchdog system overview

The Watchdog system was previously tested using clinical dynamic IMRT fields delivered to an anthropomorphic prostate phantom [13] and was implemented for clinical use [14]. Predicted EPID images were calculated for sequential dose increments using the physics-based model of Chytyk et al. [18, 19]. The predicted EPID images were generated at predetermined control point (CP) intervals for each treatment plan file, providing a sequence of frames for the entire beam delivery, as described in [13, 14]. For IMRT deliveries a synchronization method using MLC leaf positions extracted from predicted and measured images is applied [13]. The system was used to compare cumulative predicted and integrated measured frames up to a synchronization point (referred to as a cumulative image comparison). The measured images were resized to ½ resolution (512 × 384 pixels) and 2-D comparisons were evaluated by using a fast χ comparison with 4 %, 4 mm criteria [20]. The system achieved a mean real-time χ pass rate of 91.1 % for 4 %/4 mm criteria [14]. The framework of the Watchdog system is summarised and illustrated in Fig. 1. After acquisition and analysis the predicted and measured EPID images are stored in a database as well as the real-time verification result and any manually entered comments by the operator.

### Statistical process control for deriving control limits

#### Determination of lower control limit (LCL)

The application of SPC control limits assists in the classification of normal and assignable (special) causes of variation in a process based on training data [21]. The limits for this classification are set by calculating the mean(μ) and standard deviation(σ) of a process metric when the process is under stable operation. SPC normally employs two statistical control limits and a centre line (CL), including an upper control limit (UCL) and a lower control limit (LCL). However, in this application the real-time verification output (i.e. χ comparison pass-rate) cannot exceed 100 % so the UCL is eliminated and only the LCL is considered.

As the linac dose-rate increases rapidly after beam-on, the EPID central axis dose response increases rapidly often with a small overshoot, and then stabilises after approximately two seconds [22, 23]. To avoid this region of dose rate instability, the method used to calculate control limits excluded the first two seconds of treatment. CLs and LCLs were determined for the cumulative χ pass rate for three treatment sites; prostate, HN, and rectum using Eqs. 1 and 2.

1 $$ C{L}_{t\ge 2s}=\kern0.5em {\mu}_{t\ge 2s}, $$

2 $$ LC{L}_{t\ge 2s}=\kern0.5em {\mu}_{t\ge 2s}\kern0.5em -3{\sigma}_{t\ge 2s}, $$

Where ${\mu}_{t\ge 2s}$ is the average cumulative dose comparison pass-rate after two seconds of treatment, and ${\sigma}_{t\ge 2s}$ is the standard deviation of cumulative dose comparison pass-rate after two seconds. After that, the control chart is constructed based on the defined LCL for the specific treatment site.

#### Error detection

Monitoring using LCL alone however is sensitive to highly transient events and therefore a second parameter was introduced to provide improved identification of clinically significant errors. The second parameter uses a process evaluation technique to assist in error detection.

The process evaluation uses a process capability index $\left({C}_{pml}\right)$, which represents the ability of a process to produce data that meet the LCL. Using the training data set, the process capability index is calculated using Eq. 3.

3 $$ {C}_{pml-t}=\frac{\mu_{t\ge 2s}-LCL}{1.46\sqrt{{\sigma_{t\ge 2s}}^2+{\left({\mu}_{t\ge 2s}-T\right)}^2}} $$

Where ${\mu}_t$ and ${\sigma}_t$ are the average and the standard deviation of cumulative dose comparison pass-rate after two seconds of treatment up to the delivery point. The constant 1.46 is recommended for a one sided specification limit [24] and T is the process target value that can be assumed to be the average of the cumulative dose comparison pass-rate or the centre line shown in Eq. 1 [25]. Note that, there is no single ideal value of ${C}_{pml}$ that ensures a process is operating optimally. Typically ${C}_{pml}=1.33$ is used as a lower limit for an acceptably performing process and indicates a high quality QA process [26].

The delivery was classified as a “fail” when both of the following two conditions were true:

1. The real-time cumulative χ pass rate was less than the site-specific LCL
2. The process capability index, ${C}_{pml}$ was less than 1.33

### Sensitivity testing

The sensitivity of the derived control limits to various sources of error was tested and evaluated. This was performed using two prostate patient data sets. Simulated error classes were introduced by modifying the patient CT scan data or treatment plan parameters, then re-calculating the predicted EPID image set. Table 1 presents the list of test cases and simulation parameters used for the sensitivity testing. A comparison between the predicted and measured transit images was then performed using an offline simulator of the real-time verification system.

**Table 1:** Sensitivity test cases and modified parameters for introducing errors into prediction model

Error Class	Simulated Errors	Plan modification methods
Patient position misalignment	Patient setup errors = 5, 7, 10 mm	Horizontal spatial shift of CT images data set, then recalculate predicted EPID data; only gantry angle at zero is tested.
MU errors	Increasing 5, 7, 10 %	Decrease MU in treatment plan then calculate predicted EPID data; this simulates overdose delivery to patient.
Wrong Patient or Plan	Incorrect patient same treatment site	Apply different patient CT data for predicted EPID data. This simulates wrong patient being treated.
Wrong gantry angle	Correct plan (patient) incorrect field or wrong gantry angle	Re-ordered gantry angle in treatment plan (exchanged gantry angle between treatment fields) then re-calculated predicted EPID data. This simulates wrong gantry angle in IMRT.

### Classification of error sources

Five randomly selected patients for prostate, HN, and rectum IMRT treatments were used for patient treatment evaluation in this preliminary investigation. The derived site-specific control limits were used to examine and classify the clinical real-time verification results with the selected patient data set including all fractions from each treatment site. A “fail” is triggered when the individual treatment evaluation exceeds the condition of error detection (cumulative χ comparison < LCL and C*pml* < 1.33). Any failure during the verification prompted further analysis to classify and determine the source of the error.

The capability of error detection using the system was investigated and classified as one of two main categories; clinical and Watchdog system related sources [27]. For clinical error sources, there are two subcategories; patient related delivery errors and data transfer and linac errors. Three subcategories are related to non-clinical error sources, including EPID acquisition errors, Watchdog system errors, and Watchdog user errors. Observation parameters are presented in Table 2.

**Table 2:** Overview of categories of errors sources for the system and observation parameters for error classification

Error category	Clinical error (Y/N)?	Observation Parameters	Error sources
Acquisition error	N	Number acquisition frames/average EPID pixel offset/RTT comments	Errors during acquisition of EPID images (i.e. missing frames, incorrect EPID calibration)
Watchdog system error	N	Synchronisation results/Difference between integrated predicted VS measured EPID and integrated measured EPID VS measured EPID in different fractions	Errors caused by incorrect synchronization between predicted and measured EPID image and inaccuracy of predicted EPID image calculation
Watchdog user error	N	RTT comments/Number acquisition frames/Predicted EPID plan information VS patient information	Errors made by RTTs using Watchdog software (e.g. started Watchdog software after beam-on, selecting wrong predicted plan)
Transfer and machine error	Y	MLC position and trajectory [28]/Gantry angle/Collimator angle [13]/Treatment information on image header/Beam profile calculation	Errors during data transfer from TPS to treatment equipment and due to malfunctioning of treatment machine (e.g. erroneous field sizes, number of monitor units or collimator angles entered into the treatment machine, wrong MLC leaf positions or trajectory)
Patient related delivery error	Y	Beam profile/Visual assessment of predicted and measured EPID images/Normalised dose comparison/RTTs comment	Errors caused by set-up errors or intra- and inter-fractional organ motion and due to inaccuracies during the individualised treatment (e.g. occurrence of gas pockets in the rectum, patient weight loss, and erroneous density correction in TPS).

## Results

### Treatment site-specific LCLs

The derived lower control limits of prostate, HN, and rectum IMRT were 75.6, 71.3, and 71.1 % respectively (see Table 3). The average cumulative χ pass-rate percentage or centre line were 89.1, 84.0, and 80.9 % for prostate, HN, and rectum IMRT.

**Table 3:** Results of treatment site-specific lower control limits

Treatment type	Treatment site	Number of plans or patients	Number of fields	Average cumulative χ pass-rate (%) (μ)	Lower control limit (%) (μ-3σ)
IMRT	Prostate	82	1055	89.1	75.6
IMRT	Head and Neck	37	486	84.0	71.3
IMRT	Rectum	18	259	80.9	71.1

### Sensitivity testing

Table 4 presents the sensitivity test results with the testing parameters and detection delay. The results of the sensitivity testing showed that the system is able to detect 5 % MU error, wrong patient, and wrong gantry angle, however it was not sensitive to small patient displacements, except in one field with 10 mm displacement. Because the sliding window IMRT field aperture characteristically starts from left and moves to the right this means the system can exhibit a delay in detecting dose delivery or patient setup errors depending on the simulated error. Note that the sensitivity may vary based on the patient anatomy, treatment field size, shape, gantry angle, and MLC speed. The error detection for wrong dose delivery (MU error) is more sensitive than setup error. For a 5 % absolute dose error the system detected the error after about 23 % of treatment had been delivered. For the gross errors including wrong patient and wrong gantry angle the system could detect these immediately after 2 s or after about 15 % of the treatment had been delivered.

**Table 4:** Sensitivity test of four test cases

Sensitivity test cases	Error parameters	# test fields	# error detection	^a^Detection delay ± 1SD (seconds)
Wrong dose delivery (MU error)	+5 %	14	14	4.6 ± 1.5
	+7 %	14	14	3.3 ± 0.9
	+10 %	14	14	2.5 ± 0.6
Patient setup errors	Shift 5 mm	6	0	N/A
	Shift 7 mm	6	0	N/A
	Shift 10 mm	6	1	8.7 ± 0.0
Wrong patient treatment	Incorrect plan, same treatment site	10	10	Immediately detected after 2 s
Wrong gantry angle	Correct plan, incorrect field/gantry angle	10	10	Immediately detected after 2 s

^a^Detection delay is the period from the start of treatment to the time that the system is able to detect the simulated errors. The system was designed not to take the first 2s into account; therefore, the detection delay must greater than 2s

### Classification of error sources

**Fig. 2:**
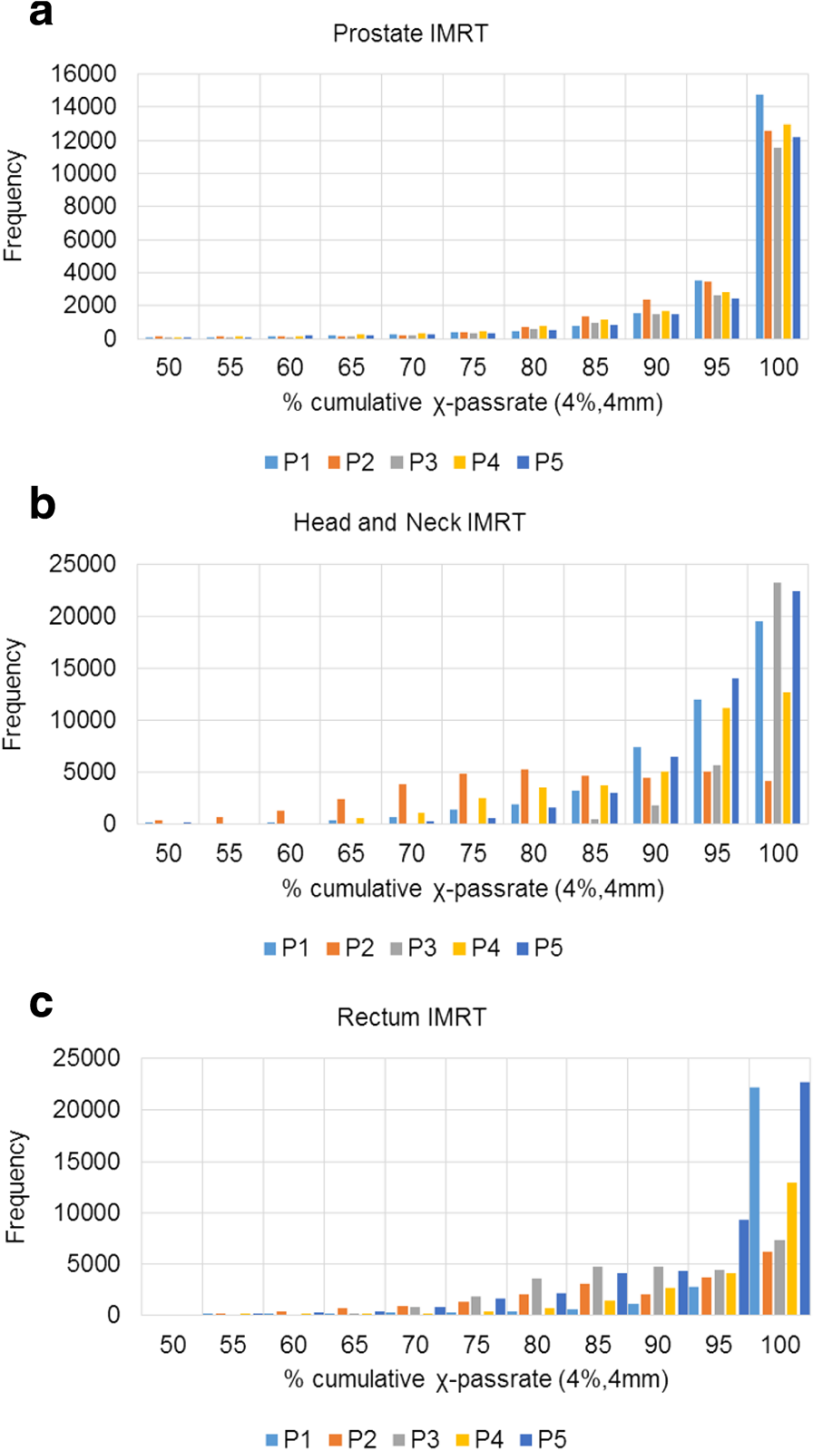
Histogram of cumulative χ comparison pass- rate for five patients studied for prostate (**a**), head and Neck (**b**), and rectum (**c**)

Figure 2 presents the histogram of the cumulative χ comparison pass-rate (4 %, 4 mm) for each treatment site. The average cumulative χ comparison pass-rates across five patients for each site were 90.5, 87.3, and 87.3 %, for prostate, head and neck, and rectum respectively. The treatment verification results were evaluated using our defined control limits and are illustrated in Table 5. The average percentage of errors detected under the condition of error detection for all patients was 7.0 % (Prostate = 5.6 %, HN = 8.7 % and Rectum = 6.7 %).

**Table 5:** Results of error detection and classification

Treatment site	Patient no.	Number of fractions operated with Watchdog	Overall % under control limit	% Under condition of error detection				
				Clinical		Non-clinical		
				Patient related delivery errors	Transfer and machine errors	Watchdog system errors	Acquisition errors	Watchdog user errors
Prostate	1	31	4.4 %	1.9 %	0.0 %	1.5 %	0.3 %	0.8 %
	2	30	6.9 %	3.7 %	0.0 %	1.8 %	0.1 %	1.2 %
	3	30	4.9 %	1.7 %	0.0 %	2.7 %	0.1 %	0.5 %
	4	35	6.1 %	2.5 %	0.0 %	2.1 %	0.3 %	1.3 %
	5	31	5.6 %	1.6 %	0.0 %	3.4 %	0.1 %	0.4 %
Head and neck	1	29	2.9 %	1.8 %	0.0 %	0.6 %	0.3 %	0.2 %
	2	32	23.7 %	11.2 %	0.0 %	8.5 %	2.5 %	1.5 %
	3	33	0.0 %	0.0 %	0.0 %	0.0 %	0.0 %	0.0 %
	4	27	7.1 %	0.9 %	0.0 %	4.7 %	0.1 %	1.4 %
	5	28	9.7 %	6.8 %	0.0 %	1.4 %	0.6 %	0.8 %
Rectum	1	20	0.0 %	0.0 %	0.0 %	0.0 %	0.0 %	0.0 %
	2	14	13.3 %	7.9 %	0.0 %	2.0 %	1.8 %	1.6 %
	3	25	4.9 %	1.3 %	0.0 %	1.8 %	0.2 %	1.6 %
	4	24	7.0 %	1.3 %	0.0 %	3.8 %	0.2 %	1.6 %
	5	29	8.4 %	4.3 %	0.0 %	2.9 %	0.1 %	1.2 %

**Fig. 3:**
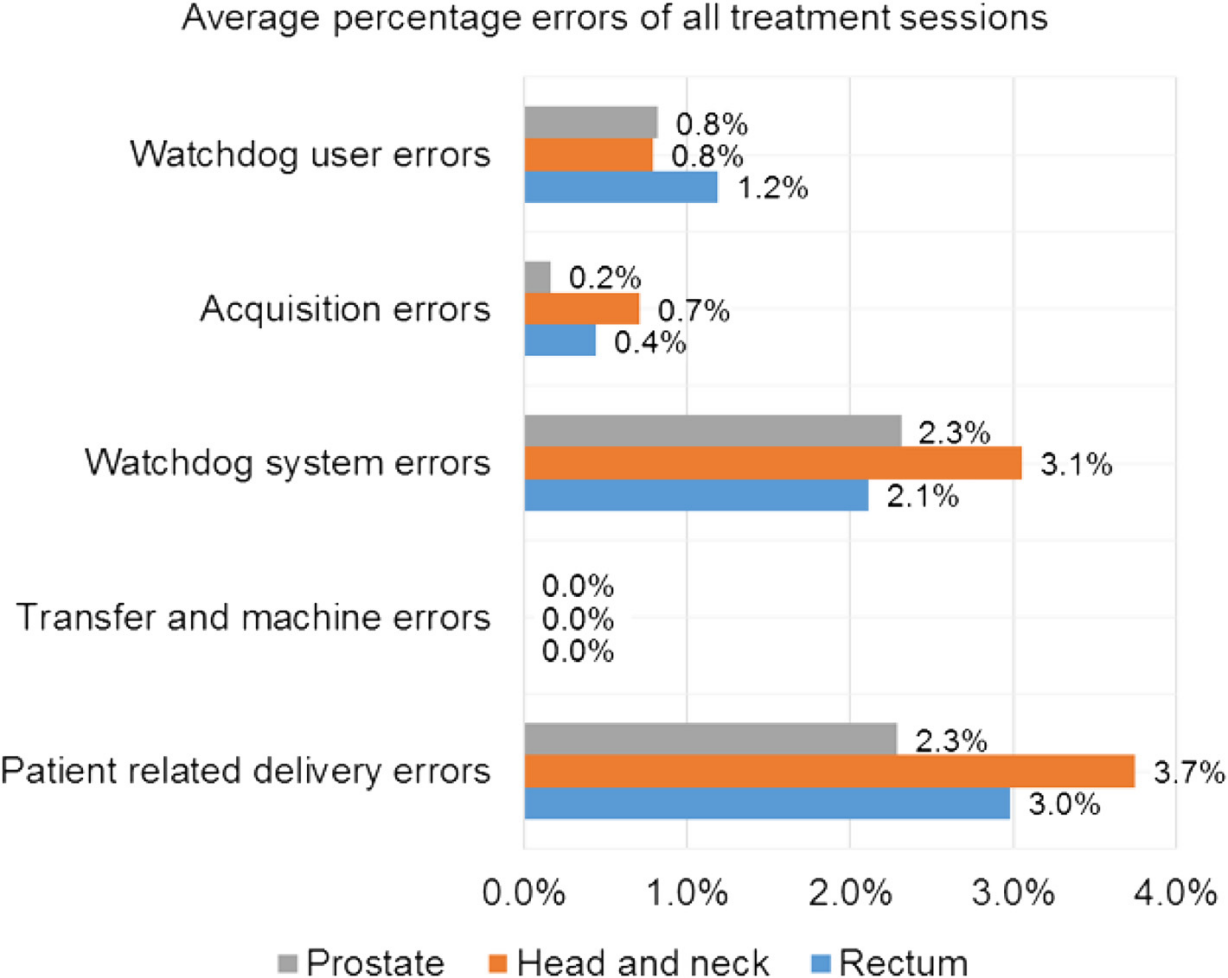
Distribution of error sources found for prostate, head and neck, and rectum using Watchdog and the observation parameters from Table 2

Individual patients were investigated to determine the source of errors using the parameters described in Table 2. Only treatments where an error was detected were evaluated (see Table 5 and Fig. 3). The average of the clinical errors was 2.3, 4.2, 3.0 % for prostate, HN, and rectum IMRT treatments respectively. In addition, the average of non-clinical errors was 3.3, 4.6, and 3.8 % for prostate, HN, and rectum IMRT treatments. There is a large spread of detected errors between individual patients for the HN and rectum cases (Fig. 2) reflecting the increased field sizes and greater complexity of field shape and anatomy in the treatment field compared to the prostate cases. Figure 3 shows the distribution of error sources found using Watchdog and our predefined action limits.

## Discussion

The real-time treatment verification system described in this work has been clinically implemented; however control limits were not determined and real-time intervention was not previously performed [14]. The initial data collection is used here to determine initial site-specific control limits and examine the performance and limitations of the system. The SPC method requires good quality data i.e. without large errors in order to determine control limits. In this study, the first two fractions from 137 patients were analysed to determine the LCL (see Table 3). The selected fractions were reviewed to ensure that there were no Watchdog system related errors, and a low likelihood of large clinical errors. However, small clinical and non-clinical random errors may be embedded into the training data sets. Therefore these LCLs will not detect such errors that may occur during clinical treatment delivery.

LCL determination requires consideration of the number of training data sets and quality of these data sets. While the numbers of patients were limited especially for rectum, the results of LCL were similar across the three treatment sites. Note that, the dose comparison criteria can be changed from 4 %, 4 mm but then the LCL would require adaptation. For example, if the criteria were 3 %, 3 mm the LCL would be decreased. Thus, changing the dose comparison criteria does not affect the proposed evaluation method. The use of different dose comparison criteria was demonstrated and discussed by Woodruff et al. [14].

**Fig. 4:**
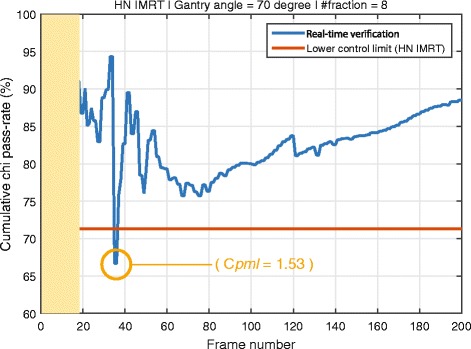
Demonstration of a real patient case of EPID synchronisation issue in HN IMRT

The error detection is based on two conditions; cumulative χ pass-rate and the process capability index $\left({C}_{pml}\right)$ value at frame *n*. Currently the cumulative χ pass-rate cannot be used alone as it shows a high sensitivity to transient fluctuations due to synchronisation errors and Watchdog acquisition errors. For example, Fig. 4 demonstrates a synchronisation error causing a significant drop in χ pass-rate at frame 35. However, the final aim is to use the χ pass-rate on its own once the system robustness has been improved through further software development.

Approximately half of detected errors came from non-clinical sources (55 % of detected errors). These error types include user errors such as failing to initiate image acquisition, image frame acquisition errors and Watchdog system errors (See Table 2). The system was found to be very sensitive to EPID calibration. This suggests that the QA of the EPID is important and verification of this response should be built into daily morning checks before treatment. The main source of Watchdog system error arises from loss of synchronisation between predicted and measured EPID images. With IMRT fields on the Varian Clinacs, synchronisation is based on an MLC aperture comparison method. Currently for VMAT the synchronisation is via the gantry angle in the image header. The false positive error detection rate is currently too high for real-time intervention, i.e. the specificity of the system must be improved. This is currently being addressed by development of more robust software engineering, improved synchronisation methods, and incorporating system self-checks into the software.

For the clinical errors (45 % of detected errors), all were caused by patient related delivery errors and no errors resulted from data transfer or linac delivery errors. These included day-to-day variations in anatomy, tumour shrinkage, intra-fractional motion, patient set-up, and patient weight loss. In several of the HN IMRT patient treatments, Watchdog was able to detect patient weight loss. The site-specific control limits identified patient related errors occurring in 2-4 % of treatments.

The Watchdog system is designed to detect major mistreatments in radiation therapy before substantial dose has been delivered to the patient. This requires real-time comparison of measured and planned delivery. As demonstrated here with these simulations, smaller errors may also be detectable with the system and SPC derived limits, however these are likely to be detected later in the delivery and would be more suited to off-line analysis. While SPC based limits were used here to investigate the system performance, separate action limits for treatment intervention will likely be required. For this further investigation is underway to determine appropriate action limits for real-time intervention based on clinical significance of the errors.

## Conclusion

A real-time error detection method using statistical process control for real-time EPID based delivery verification during dynamic IMRT has been developed and tested with patient treatment data in this work. The selected two fractions from each treatment course of 137 patients have been used to calculate the site specific lower control limit (LCL). The combination of cumulative χ comparison (less than treatment site-specific LCL) and process capability index (less than 1.33) is used to develop the real-time error detection threshold. This method was tested for sensitivity to errors using simulations by either plan or CT data modification. Manual investigation of patient detected errors was able to distinguish the errors as either clinical or non-clinical. Future work includes the determination of action limits based on clinical significance of the errors, the determination of action limits for VMAT treatments and the development of a workflow to integrate the Watchdog system into routine clinical use.
